# Rational Combinations of Targeted Agents in AML

**DOI:** 10.3390/jcm4040634

**Published:** 2015-04-10

**Authors:** Prithviraj Bose, Steven Grant

**Affiliations:** 1 Department of Internal Medicine, Virginia Commonwealth University and VCU Massey Cancer Center Center, 1201 E Marshall St, MMEC 11-213, P.O. Box 980070, Richmond, VA 23298, USA; E-Mail: pbose@mcvh-vcu.edu; 2 Departments of Internal Medicine, Microbiology and Immunology, Biochemistry and Molecular Biology, Human and Molecular Genetics and the Institute for Molecular Medicine, Virginia Commonwealth University and VCU Massey Cancer Center, 401 College St, P.O. Box 980035, Richmond, VA 23298, USA

**Keywords:** AML, targeted therapies, rational combinations, HDAC inhibitors, CDK inhibitors, proteasome inhibitors, checkpoint abrogators, apoptosis, BH3-mimetics, Mcl-1

## Abstract

Despite modest improvements in survival over the last several decades, the treatment of AML continues to present a formidable challenge. Most patients are elderly, and these individuals, as well as those with secondary, therapy-related, or relapsed/refractory AML, are particularly difficult to treat, owing to both aggressive disease biology and the high toxicity of current chemotherapeutic regimens. It has become increasingly apparent in recent years that coordinated interruption of cooperative survival signaling pathways in malignant cells is necessary for optimal therapeutic results. The modest efficacy of monotherapy with both cytotoxic and targeted agents in AML testifies to this. As the complex biology of AML continues to be elucidated, many “synthetic lethal” strategies involving rational combinations of targeted agents have been developed. Unfortunately, relatively few of these have been tested clinically, although there is growing interest in this area. In this article, the preclinical and, where available, clinical data on some of the most promising rational combinations of targeted agents in AML are summarized. While new molecules should continue to be combined with conventional genotoxic drugs of proven efficacy, there is perhaps a need to rethink traditional philosophies of clinical trial development and regulatory approval with a focus on mechanism-based, synergistic strategies.

## 1. Introduction

Despite significant progress in recent years in unraveling the genetic basis of AML [1], resulting in improvements in our ability to prognosticate and predict outcomes with certain therapies [2], it remains a devastating disease. Cure rates for young adults remain 40%–45% at best, and those for patients older than 60 only around 10%–20% [3]. The anthracycline-cytarabine backbone, first introduced over 40 years ago [4], remains the cornerstone of initial therapy for most patients, and the only truly targeted agent to receive regulatory approval, gemtuzumab ozogamycin, has been voluntarily withdrawn from the market by the manufacturer [5,6]. Allogeneic hematopoietic stem cell transplantation (HSCT), with all its attendant risks, remains the best post-remission therapy for AML till date [7]. In a recent randomized, phase III trial [8], elacytarabine, a novel elaidic acid ester of cytarabine, failed to improve outcomes over physicians’ choice of one of seven different commonly used salvage regimens for patients with relapsed or refractory disease, who have a dismal prognosis (5-year overall survival from first relapse approximately 10%) [9]. Although initially greeted with considerable enthusiasm, no fms-like tyrosine kinase 3 (FLT3) inhibitor has been licensed for use [10], and resistance-conferring mutations in the FLT3 kinase have been described [11]. Recent approaches have involved exploring new therapeutic targets, e.g., isocitrate dehydrogenase (IDH) [12] or immune checkpoints, delivering conventional cytotoxic agents in fixed molar ratios [13,14], harnessing the power of T-cells against AML stem cell antigens (e.g., CD123) using dual affinity retargeting molecules (DARTs, *Blood* 2013; 122:360), *etc.*

Malignant cells are particularly vulnerable to the simultaneous disruption of multiple, cooperative survival signaling pathways [15,16]. In recent years, the concept of rationally combining targeted agents to defeat the redundancy of survival pathways in neoplastic cells has rapidly been gaining ground in a variety of tumor types [17]. Indeed, signaling pathways within cancer cells have been compared to other complex networks such as the internet or airplane flight patterns, characterized by both remarkable robustness and surprising vulnerability, such that very limited yet coordinated, specific targeting of the most critical “nodes” in the network can have dramatically outsized effects [18]. In this article, we review various “synthetic lethal” strategies using rational combinations of targeted drugs in AML.

## 2. Combinations Involving Epigenetic Therapies

### 2.1. DNMTIs + HDACIs

A particularly popular combination has been that of DNA methyltransferase inhibitors (DNMTIs) with histone deacetylase inhibitors (HDACIs). That epigenetic processes play a fundamental role in cancer causation and progression has been recognized for over a decade now [19]. Chromatin organization modulates gene transcription inasmuch as a more open, relaxed configuration of chromatin (e.g., induced by HDACIs through histone acetylation) or demethylation of CpG islands in the promoter regions of genes (e.g., induced by DNMTIs, also known as hypomethylating agents, HMAs) activates transcription of epigenetically silenced tumor suppressor genes, e.g., DNA repair genes [20]. The combination of DNMTIs and HDACIs synergistically triggers apoptosis and up-regulates microRNAs that, in turn, down-regulate oncogenes [21]. While the DNMTIs azacytidine and decitabine have clear single-agent activity in AML [22,23,24,25] and are widely used, response rates to HDACI monotherapy in AML and MDS have been more modest, of the order of 10%–20% [26] and none is approved for this indication, although the pan-HDACI pracinostat was recently granted “orphan drug” status by the FDA for AML [27]. As noted above, many trials of “dual epigenetic therapy” combining DNMTIs with HDACIs in AML have been conducted. In the recently published U.S. Leukemia Intergroup trial E1905 involving 97 patients with MDS and 52 with AML, addition of the class I-selective HDACI entinostat to azacytidine did not increase clinical response rates and was associated with pharmacodynamic antagonism [28]. Azacytidine was administered on days 1–10 of a 28-day cycle and entinostat on days 3 and 10 [28]. A randomized phase II study (NCT01305499) is currently underway to see if sequential, as opposed to concurrent, administration of entinostat will improve efficacy of this combination. In contrast, the combination of pracinostat with azacytidine yielded an 89% overall response rate (ORR) in a 9-patient pilot study in patients with higher risk categories of MDS and was very well tolerated [29].

### 2.2. Other DNMTI-Based Combinations

DNMTIs have also been combined with many other classes of targeted agents in AML. Based on the ability of the proteasome inhibitor bortezomib to up-regulate miR-29b, resulting in loss of transcriptional activation of several genes relevant to myeloid leukemogenesis, including DNA methyltransferases and receptor tyrosine kinases, a phase I trial of bortezomib and decitabine was conducted [30]. Seven of 19 patients overall achieved complete remission (CR) or complete remission with incomplete count recovery (CRi), although 5 of these were treatment-naïve [30]. The regimen was shown to down-regulate FLT3 [30]. These findings led to a phase II trial (NCT01420926) in the cooperative group setting in older patients with AML, but this trial was closed prematurely as it was deemed unlikely to meet its primary endpoint. In a phase II trial of sorafenib and azacytidine in 43 patients (37 evaluable) with *FLT3*-mutated AML and 0–7 (median 2) prior therapies, the ORR was 46%, including 27% CR/CRi [31]. FLT3 ligand levels did not rise to levels seen in prior studies of patients receiving cytotoxic chemotherapy [32]. Decitabine and midostaurin have been combined in a phase I study in patients with relapsed/refractory AML with or without *FLT3* mutations based on *in vitro* evidence of synergy against FLT3-internal tandem duplication (*FLT3-ITD*^+^) cells [33]. Sequential administration was safe but concurrent administration was too toxic [33]. 57% of patients achieved stable disease (SD) or better; 25% had a complete hematologic response (CHR) [33]. Phase I trials combining decitabine with rapamycin [34] and with bexarotene [35] in patients with AML have been completed, with demonstration of safety but only modest outcomes. Both sequential [36] and concomitant [37] administration of azacytidine and lenalidomide have been studied in newly diagnosed elderly patients with AML in the phase I setting. Both combinations were well-tolerated, with ORRs of around 55% and CR/CRi rates ranging from 31% to 44% [36,37].

Given their efficacy and safety, and consequent widespread use as single agents for the treatment of patients with AML who are elderly and/or unfit for conventional chemotherapy, azacytidine and decitabine are increasingly being combined in clinical trials with a plethora of emerging, investigational targeted agents. Examples include volasertib (NCT02003573), a polo-like kinase 1 (PLK-1) inhibitor recently designated an “orphan drug” for AML [27], the first-in-class neddylation inhibitor MLN4924 (NCT01814826), the hedgehog inhibitor sonidegib (NCT02129101) and the Bcl-2-selective antagonist, ABT-199 (NCT02203773). *In vitro*, however, short interfering RNA (siRNA) silencing of the anti-apoptotic proteins Mcl-1 and Bcl-xL, but not Bcl-2, exhibited variable synergy with azacytidine [38].

### 2.3. BET Inhibitor-Based Combinations

A relatively recent development in the field of epigenetic targeting of chromatin networks in cancer has been the discovery of potent and specific small-molecule BET (bromodomain and extraterminal) inhibitors [39]. The bromodomain is a highly conserved motif of 110 amino acids found in proteins that interact with chromatin, such as transcription factors, histone acetylases and nucleosome remodelling complexes [40]. Bromodomain proteins function as chromatin “readers”, some of which have key roles in the acetylation-dependent assembly of transcriptional regulator complexes [41]. Bromodomain proteins recruit chromatin-regulating enzymes, including “writers” and “erasers” of histone modification, to target promoters and to regulate gene expression [40]. Lysine acetylation, a key mechanism that regulates chromatin structure, creates docking sites for bromodomains, and BET proteins regulate the expression of key oncogenes and anti-apoptotic proteins [41]. It was recently demonstrated that the BET protein (BRD4) antagonist JQ1 synergistically induced apoptosis of AML cells in combination with the HDACI panobinostat and improved the survival of mouse xenografts [42]. Additionally, JQ1 also synergized with the FLT3 tyrosine kinase inhibitors (TKIs) ponatinib or quizartinib to induce apoptosis of *FLT3-ITD*^+^ AML cells and overcame FLT3 TKI resistance-conferring mutations such as F691L and D835V [43]. Furthermore, the JQ1/panobinostat combination synergistically induced apoptosis of FLT3 TKI-resistant cells [43].

## 3. HDACI-Based Combinations Involving Non-Epigenetic Therapies (Table 1)

**Figure 1:**
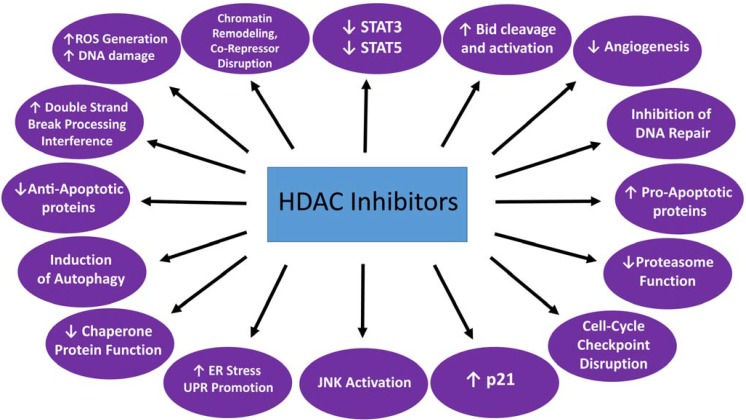
Mechanisms of HDACI lethality. Reproduced, with permission, from [45].

Distinct from their role as epigenetic modifiers, HDACIs exert a plethora of other actions (Figure 1) in neoplastic cells with a high degree of selectivity for the latter. These include down-regulation of anti-apoptotic and up-regulation of pro-apoptotic proteins (e.g., Bim), activation of the death receptor (extrinsic) pathway of apoptosis, induction of oxidative injury, interference with checkpoint and chaperone protein function (the latter through acetylation of Hsp90, leading to down-regulation of its “client” proteins), inhibition of DNA repair, interference with the function of co-repressors/co-factors, promotion of endoplasmic reticulum (ER) stress and disruption of aggresome function, JNK (C-Jun-N-terminal kinase) activation, STAT5 (signal transducer and activator of transcription 5) inhibition, proteasome inhibition, induction of autophagy and anti-angiogenic effects [26,44,45].

### 3.1. HDACIs + Proteasome Inhibitors

As noted above, the single-agent activity of HDACIs in AML is modest [26], and it has been appreciated for some time that the ultimate role of these agents may lie in combinatorial approaches [44,46]. An extensively studied rational combination has been that of HDACIs with proteasome inhibitors (PIs). While most advanced in multiple myeloma (MM) in terms of clinical development [47], this combination has been investigated in nearly every hematologic malignancy and may hold promise in AML [48,49]. Synergism between PIs and HDACIs stems from multiple mechanisms [50], including the inhibition by PIs of the pro-survival NF-κB pathway [51], which is activated by HDACIs and limits their lethality [52,53], disruption by HDACIs of aggresome formation [54], a physiologic response to proteasome inhibition [54], Hsp90 inhibition by HDACIs [55], both leading to marked accumulation of mis-folded proteins and accentuation of the proteotoxic stress induced by PIs, and multiple overlapping actions between these two classes of agents [26,44,45,56,57]. Additionally, there is evidence that the proteasome plays an important role in HDACI-induced apoptosis [58], and that HDACs are critical targets of bortezomib, at least in MM [59].

In pre-clinical studies, co-administration of sub-micromolar concentrations of the pan-HDACI belinostat with low nanomolar concentrations of bortezomib sharply increased apoptosis in AML and cell lines and primary blasts [48]. Synergistic interactions were associated with interruption of both canonical and non-canonical NF-κB signaling pathways, down-regulation of NF-κB-dependent pro-survival proteins (e.g., XIAP, Bcl-xL) and up-regulation of Bim [48]. These findings led to a phase I clinical trial of the combination in patients with relapsed/refractory or poor-prognosis previously untreated acute leukemias or higher risk MDS (NCT01075425) [49]. The maximum tolerated doses (MTDs) were determined to be 1.3 mg/m^2^ IV of bortezomib on days 1, 4, 8 and 11 and 1000 mg/m^2^ IV of belinostat on days 1–5 and 8–12 of a 3-week cycle. Of 35 response-evaluable subjects, one patient with mixed lineage leukemia (*MLL*)-rearranged biphenotypic acute leukemia refractory to “7 + 3” and FLAG-Ida (fludarabine, cytarabine, granulocyte colony stimulating factor, idarubicin) achieved a CR and went on to an allogeneic HSCT; 4 had a partial remission (PR), 14 achieved stable disease (SD), and 16 had disease progression. Four subjects discontinued study treatment due to adverse events (AEs). Overall, the regimen was very well-tolerated. One patient with Janus kinase 2 (*JAK2*)-mutated myelofibrosis transformed to AML remains on treatment beyond 31 cycles (manuscript in preparation). Efforts are currently underway to better characterize (at a genomic level) the leukemias of the two patients who did exceptionally well on this study.

### 3.2. HDACIs + CDK Inhibitors

Cyclin-dependent kinase inhibitors (CDKIs) represent an interesting class of agents capable of inducing cell cycle arrest and apoptosis in malignant cells, particularly hematologic tumor types, which may be more susceptible to inhibition of cell cycling and apoptosis induction [60]. Some of these agents, *i.e.*, those that inhibit cyclin T/CDK9, additionally inhibit global cellular transcription [61], thus down-regulating short-lived proteins critically dependent on transcription for their maintenance, e.g., the anti-apoptotic proteins XIAP and Mcl-1. Although no CDKI is currently approved, the FDA recently granted “orphan drug” designation to the pan-CDKI flavopiridol (alvocidib) for AML [27].

Multiple pre-clinical studies have demonstrated robust synergism in AML cells between pan-CDKIs such as flavopiridol or roscovitine and HDACIs, e.g., vorinostat, dacinostat, sodium butyrate [62,63,64,65,66]. Besides down-regulation of XIAP and Mcl-1, a major mechanism underlying these interactions was the blockade by CDKIs of HDACI-induced up-regulation of the endogenous CDKI, p21^WAF1/CIP1^. These observations led to a phase I trial of the combination of alvocidib and vorinostat in patients with relapsed/refractory or poor prognosis newly diagnosed acute leukemia or higher risk MDS [67]. The alvocidib MTD was 20 mg/m^2^ IV load over 30 min followed by 20 mg/m^2^ infused over 4 h (“hybrid” schedule of administration [68]) on days 1 and 8, in combination with vorinostat, 200 mg orally, three times a day, for 14 days on a 21-day cycle [67]. No objective responses were achieved in 26 evaluable patients (of 28 treated), although 13 exhibited SD [67].

### 3.3. HDACIs + TKIs

As discussed above, HDACIs, especially those that inhibit HDAC6, acetylate and thereby interfere with the function of chaperone proteins, in particular Hsp90, consequently down-regulating several pro-growth and pro-survival Hsp90 “client” proteins of critical importance in myeloid leukemias, e.g., breakpoint cluster region-Abelson (Bcr-Abl), mutant FLT3, c-Raf and Akt [55]. In the context of FLT3, this phenomenon has also been reported with the class I-selective HDACI, entinostat [69]. Additionally, HDACIs disrupt mitotic spindle checkpoints in neoplastic cells [70] and induce “mitotic slippage” [71]. These findings have provided the rationale for multiple preclinical studies that have demonstrated the synergism between HDACIs and TKIs targeting Bcr-Abl [72,73,74,75,76], FLT3 [77], JAK [78,79] and aurora kinases [80,81,82], which play critical roles in mitosis [83,84]. Unfortunately, the dual Bcr-Abl/aurora kinase inhibitors MK-0457 (VX-680) [80,81] and KW-2449 [82] are no longer in development. To our knowledge, this concept has not been evaluated in clinical trials in AML. However, at least two ongoing clinical trials are testing the combination of the HDACI panobinostat and the JAK1/2 inhibitor ruxolitinib in patients with myelofibrosis (NCT01693601, NCT01433445). In chronic myeloid leukemia (CML), the HDACI+TKI strategy has been reported to target stem cells [85]. Synergistic anti-leukemic interactions between AT9283, a multi-targeted TKI that inhibits Bcr-Abl, FLT3, JAK and aurora kinases [86,87,88], and entinostat have been observed in Bcr-Abl^+^ cells, including those bearing the gatekeeper mutation T315I, as well as in AML cells, both *FLT3*-mutated and -wild type (Nguyen and Grant, unpublished observations). Considering that mutations that confer resistance to one of the most promising FLT3 TKIs, quizartinib, have been described [11], this strategy may yet prove valuable in AML.

### 3.4. HDACIs + G2/M Checkpoint Abrogators

Cell cycle checkpoints, part of the DNA damage response (DDR) network, are in-built safety mechanisms the activation of which helps preserve genomic integrity by halting cell division upon the occurrence of DNA damage and allowing time for DNA repair [89]. If repair fails, checkpoints trigger apoptosis [90]. The major cell cycle checkpoints are the G1/S, intra-S-phase and G2/M checkpoints. Checkpoint dysfunction is common in human cancers and is considered a pathologic hallmark of neoplastic transformation [91]. In particular, the G1/S-checkpoint is frequently dysfunctional because of p53 and/or Rb mutations, making malignant cells overtly reliant on the intra-S-phase and G2/M checkpoints [92]. Although p53 mutations are uncommon in de novo AML [93], overexpression of Mdm2, the negative regulator of p53, is common [94,95], as is disruption of regulated p53 expression [96], and p53 mutations are common in secondary AML [97,98,99,100]. p53 mutations are strongly associated with a complex aberrant karyotype in AML [101]. In these patients, p53 alterations are associated with older age, genomic complexity, specific DNA copy number alterations, monosomal karyotypes, and a dismal outcome [102]. When present, p53 mutations confer an extremely poor prognosis [93,103,104] that is not overcome even by allogeneic HSCT [105]. Finally, recent studies indicate that leukemic cells expressing FLT3-ITD display defective DNA repair mechanisms [106,107]. In the presence of a dysfunctional G1/S checkpoint, G2/M checkpoint abrogation (e.g., with small-molecule inhibitors of the Chk1 or Wee1 kinases) prevents cancer cells from repairing DNA damage, forcing them into a premature and lethal mitosis (“mitotic catastrophe”) [92].

Given that HDACIs induce DNA damage [108] and inhibit DNA repair, both homologous [109] and non-homologous end-joining (NHEJ) [110], and the ability of these agents and Hsp90 inhibitors to down-regulate proteins that play major roles in the DDR network such as ATR (ATM and Rad3 related), Chk1 and Wee1 [111,112,113,114], the combination of HDACIs with G2/M checkpoint abrogators has considerable theoretical appeal [45]. Indeed, synergistic potentiation of vorinostat-mediated apoptosis by the Chk1 inhibitor MK-8776 has been demonstrated in various AML cell lines, both *p53*-wild type and -deficient, as well as in those bearing *FLT3-ITD* [115]. Furthermore, the regimen was active against primary AML blasts, particularly against the putative leukemia initiating cell (LIC, CD34^+^CD38^−^CD123^+^) population [115]. However, clinical trials of Chk1 inhibitors have concentrated on combining them with conventional genotoxic agents, and no trials have explored simultaneous HDAC and Chk1 inhibition.

The Wee1 kinase has recently emerged as a novel therapeutic target in AML [116,117,118]. Although efforts at the preclinical level to develop AZD-1775, a potent, small-molecule inhibitor of Wee1 [119,120], in AML have focused largely on using it to circumvent resistance to cytarabine [121,122], a sound rationale exists for combining this agent with HDACIs in AML. Of note, AZD-1775 may be effective regardless of p53 functionality [120,122]. During interphase, Wee1 phosphorylates the cyclin B/CDK1 (also known as cdc2) complex at Tyr^15^ to inactivate it, and Wee1 inhibition causes forced activation of CDK1, premature mitotic entry and impairment of homologous recombination [123]. Activation of cyclin B/CDK1 (cdc2) requires dephosphorylation by the CDC25 phosphatases (A, B and C) [89]. Notably, inactivation of cdc2 (CDK1) involves phosphorylation at two inhibitory sites, *i.e.*, Tyr^15^ and Thr^14^, and dephosphorylation of both sites is necessary for full cyclin B/CDK1 (cdc2) activation. Upon G2/M checkpoint activation, ATR/Chk1 phosphorylates (and thereby inhibits) CDC25A, -B and -C, thus preventing premature mitotic entry [89].

While Chk1 is a positive regulator of Wee1 (through stimulatory phosphorylation), Wee1 inhibition results in compensatory activation of Chk1, leading to phosphorylation of cyclin B/CDK1 (cdc2) at Thr^14^ [124], a therapeutically undesirable, putatively cytoprotective effect. Concomitant administration of an HDACI may circumvent this problem by down-regulating Chk1 [111]. While this could also be achieved by combined Chk1 and Wee1 inhibition [124,125,126,127], HDACIs carry the additional advantages of inducing DNA damage and inhibiting DNA repair. Preclinical studies in AML with the combination of vorinostat and AZD-1775 have shown striking synergism, irrespective of *p53* and *FLT3* mutational status, including in “LIC”s, primary AML blasts and in a xenograft mouse (flank) model [128]. Importantly, whereas AZD-1775 treatment of leukemia cells triggered cyclin B/CDK1 (cdc2) Tyr^15^ dephosphorylation, it also induced Chk1 activation and Thr^14^ phosphorylation [128]. However, HDACI co-administration abrogated these undesirable phenomena, resulting in pronounced Tyr^15^ and Thr^14^ dephosphorylation, and full cyclin B/CDK1 (cdc2) activation, accompanied by premature mitotic entry and DNA damage [128]. These data and similarly promising results obtained upon substituting the recently approved HDACI belinostat for vorinostat have sparked interest in a National Cancer Institute-sponsored phase I clinical trial of the AZD-1775/belinostat combination in patients with relapsed/refractory AML/MDS as well as treatment-naïve poor prognosis patients with AML.

### 3.5. Other HDACI-Based Rational Combinations in AML

Aside from the strategies discussed above, HDACIs may potentially be successfully combined with a number of other investigational agents in AML. The first-in-class polo-like kinase 1 (PLK1) inhibitor volasertib was recently granted first “breakthrough” [129], and then “orphan drug” designation [27] for AML. This agent is currently in clinical trials in combination with low dose cytarabine (NCT01721876), decitabine (NCT02003573) or intensive chemotherapy (NCT02198482). PLK1 is critical to mitotic progression [130,131], and plays an important role in the DDR network [132,133], interacting with multiple checkpoint proteins [134]. As Bcr-Abl signals downstream to PLK1 [135], the PLK1 inhibitor BI2536 was studied in combination with vorinostat in CML cell lines and primary cells [136]. Pronounced synergism was observed in both imatinib-sensitive and -resistant Bcr-Abl^+^ cells, both *in vitro* and *in vivo* [136]. Enhanced Bcr-Abl pathway inhibition did not appear to be the predominant mechanism for lethality of the PLK1 inhibitor/HDACI combination; rather, it seemed to be potentiation of DNA damage and disabling of the DDR [136]. Given that pracinostat has also received “orphan drug” designation for AML [27], the combination of volasertib and pracinostat warrants attention in this disease.

The first-in-class inhibitor of protein “neddylation”, MLN4924 [137], is another promising agent in AML [138,139] currently in clinical trials with azacytidine (NCT01814826). Targeting protein neddylation, a critical pathway of protein degradation located upstream of the proteasome [140], allows for more selective interference with protein turnover, potentially yielding a better therapeutic index for these drugs as compared to PIs [141,142]. At least in theory, combination of this agent with HDACIs is particularly appealing for several reasons [45]. MLN4924 inhibits NF-κB (activated by HDACIs [52,53]) and leads to ROS generation and DNA damage in AML cells [138]. Additionally, MLN4924 induces DNA re-replication, an irreversible cellular insult that leads to apoptosis, by interfering with the turnover of the cullin-RING ligase substrate CDT1, a critical DNA replication licensing factor, in S-phase [143,144]. Finally, MLN4924 appears to trigger a cytoprotective autophagic response [145,146], that could be counteracted by HDACIs [147,148].

The phosphatidylinositol-3-kinase/Akt/mammalian target of rapamycin (PI3K/Akt/mTOR) pathway is a cellular growth, proliferation, motility and survival signaling axis [149] that represents one of the most frequently dysregulated pathways in cancer [150], including AML [151,152], where activation of the pathway has been shown to be required for cell survival [153,154]. In AML, Akt activation (phosphorylatyion at Thr^3^°^8^/Ser^473^) variably occurs in 50–80% of patients [155,156]; hence, there is considerable interest in targeting the PI3K/Akt/mTOR axis in AML [157]. Although mTOR inhibitors have been commercially available for some time for the treatment of various solid tumors, the first PI3K (delta isoform) inhibitor to receive regulatory approval, idelalisib [158,159], has only very recently arrived on the market, fueling intense interest in this class of agents. Synergistic interactions between PI3K or Akt inhibitors and HDACIs have been documented in AML cells [160,161]. Combined HDAC and PI3K inhibition led to a marked increase in apoptosis associated with Bcl-2 and Bid cleavage, XIAP and Mcl-1 down-regulation, mitogen activated protein kinase (MAPK) inactivation and blockade of HDACI-mediated induction of p21^CIP1/WAF1^ [160]. Inactivation of extracellular signal-regulated kinase (ERK) was also seen with HDACI/perifosine (Akt inhibitor) co-treatment of AML cells, along with Akt inhibition, JNK activation, ROS and ceramide generation, leading to striking increases in mitochondrial injury and apoptosis [161].

## 4. Priming Apoptosis

The Bcl-2 family of proteins stands at the crossroads of cellular survival and death, and the pro- and anti-apoptotic members of this family regulate the intrinsic, or mitochondrial, pathway of apoptosis [162]. The seminal event in this pathway of programmed cell death, “mitochondrial outer membrane permeabilization (MOMP)”, commits the cell to apoptosis and constitutes a “point of no return” triggered by the apoptosis “effectors” Bax and Bak [163]. Under normal conditions, the pro-survival anti-apoptotic proteins (mainly Bcl-2, Bcl-xL and Mcl-1) sequester the apoptosis effectors; thus, the latter need to be released from such binding in order to induce MOMP [163]. While some of the so-called “BH3-only” proteins (e.g., Bim, tBid and Puma) can directly activate Bax and Bak [163], most function as “sensitizers”, *i.e.*, they displace the apoptosis effectors from their association with the anti-apoptotic proteins [164], a function mimicked by the “BH3-mimetic” class of drugs. The Bcl-2 family is of profound importance in the pathogenesis, prognosis, chemoresistance and treatment of AML [165].

The discovery of ABT-737, a specific “BH3-mimetic” antagonist of Bcl-2 and Bcl-xL, demonstrated for the first time that specific protein-protein interactions could be targeted by small molecules, and ushered in a new era in cell death research [166]. Subsequently, an oral analog with improved pharmacological properties, ABT-263 (navitoclax), was developed [167]. This agent demonstrated promising efficacy in patients with relapsed/refractory CLL in a phase I trial [168], but the occurrence of dose-dependent thrombocytopenia owing to on-target Bcl-xL inhibition [169] in early clinical trials [168,170,171,172] precluded continued development of this agent. There is also some evidence that Bcl-xL-inhibitory BH3-mimetics can undermine platelet function [173]. These observations led to the development, by reverse engineering of navitoclax, of ABT-199 (GDC-0199), a highly selective Bcl-2 antagonist that retains significant anti-tumor activity while sparing platelets [174]. This agent appears highly effective in CLL [175], and at least at the preclinical level, holds substantial promise in AML [176]. Of note, oblimersen, an anti-sense oligonucleotide against Bcl-2, failed in phase III clinical trials when added to chemotherapy in older patients in AML [177], despite improving 5-year survival when combined with fludarabine and cyclophosphamide in patients with relapsed/refractory CLL [178]. This agent was not, however, ever approved for use.

**Figure 2:**
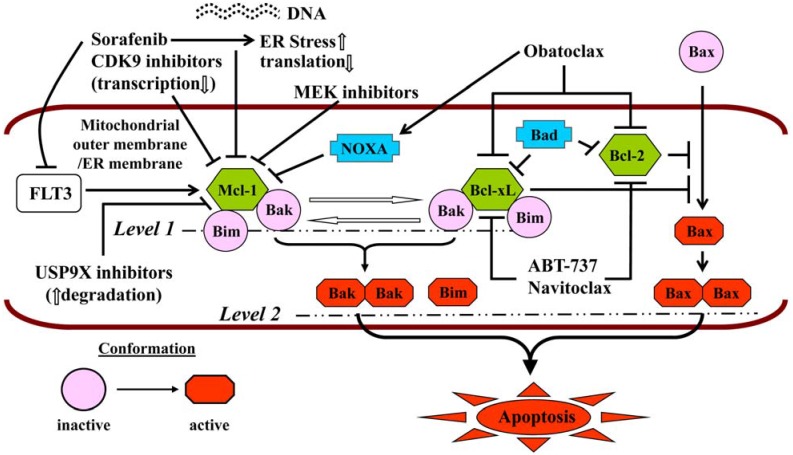
Should be: Mechanisms of potentiation of BH3-mimetic lethality by strategies targeting Mcl-1. Reproduced, with permission, from [184].

Neither ABT-199 (GDC-0199) nor navitoclax inhibits Mcl-1, which is fundamental to the pathogenesis and maintenance of AML [179] and the main determinant of resistance to ABT-737 [180,181]. The pan-BH3-mimetic obatoclax, which inhibited Mcl-1 in addition to Bcl-2/-xL [182], has been discontinued due to the occurrence of severe, infusional neurologic toxicity, e.g., ataxia, euphoria and somnolence. For these reasons, a number of combination strategies (Figure 2) have been explored preclinically to simultaneously target multiple arms of the apoptotic regulatory machinery, *i.e.*, Mcl-1 and Bcl-2/-xL [183,184]. Since Mcl-1 is a short-lived protein critically dependent on active transcription and translation for its maintenance, some of these strategies have used cyclin T/DCK9 inhibitors or sorafenib to repress cellular transcription [61] or translation [185], respectively. Thus, roscovitine dramatically increases ABT-737 lethality in AML cells by simultaneously and cooperatively inducing Bak activation and Bax translocation [186]. In the case of sorafenib, synergistic interactions with both ABT-737 [187] and obatoclax [188] have been reported, in the latter case with the induction of cytoprotective autophagy that could be inhibited pharmacologically, potentiating the lethality of the regimen [188]. In these studies, both Bim up-regulation and Mcl-1 down-regulation were noted, and synergism was demonstrated not only in AML cell lines, but also in patient-derived cells and in a xenograft mouse model [187,188]. HDACIs, which up-regulate Bim, have also been combined with both ABT-737 [189] and obatoclax [190]. HDACI-induced Bim is largely sequestered by Bcl-2 and Bcl-xL, which it is released by ABT-737, activating Bax and Bak and triggering MOMP [189]. Both mocetinostat and vorinostat display synergistic anti-leukemia activity with obatoclax, but in this setting, cell death is attributable to activation of both apoptosis and autophagy [190]. Finally, mitogen activated protein kinase kinase (MEK) inhibitors have been shown to synergize with ABT-737, both *in vitro* (including in “LICs”) and *in vivo*, through the same mechanism, *i.e.*, down-regulation of Mcl-1, which is induced by ABT-737 via ERK activation [191].

**Table 1:** HDACI-based rational combinations with non-cytotoxic, non-epigenetic agents in AML.

Partner Agent Class	Mechanism(s) of Synergy	Clinical Trials, if any	Reference(s)
Proteasome inhibitors (PIs), e.g., bortezomib, carfilzomib, ixazomib, oprozomib, marizomib	NF-κB inhibition by PIs (activated by HDACIs); inhibition by HDACIs of aggresome formation and of Hsp90→increased proteotoxic stress, multiple other actions	NCT01075425; closed to accrual; phase I; enrolled primarily relapsed/refractory patients with AML; one CR, one prolonged SD (see text)	[48,49]
Cyclin-dependent kinase inhibitors (CDKIs), e.g., flavopiridol (alvocidib), roscovitine (seliciclib), dinaciclib, palbociclib	Down-regulation of XIAP and Mcl-1 by cyclin T/CDK9 inhibitors via transcriptional repression; blockade by CDKIs of HDACI-induced up-regulation of p21	NCT00278330; completed; phase I; enrolled primarily relapsed/refractory patients with AML; no objective responses; 50% achieved SD	[62,63,64,65,66,67]
Multi-kinase inhibitors (that inhibit aurora kinases and critical signaling molecules in AML, e.g., FLT3, JAK2), e.g., MK-0457, KW-2449, AT9283	Down-regulation of Hsp90 “client” proteins by HDACIs, e.g., FLT3, c-Raf, Akt, JAK2, disruption of mitotic spindle checkpoints and induction of mitotic “slippage”		[77,79,81]
Checkpoint abrogators, e.g., MK-8776 (Chk1 inhibitor), AZD-1775 (Wee1 inhibitor)	Induction of DNA damage and inhibition of DNA repair by HDACIs; down-regulation of ATR, Chk1 and Wee1 by HDACIs via Hsp90 inhibition	Phase I clinical trial of Wee1 inhibitor AZD-1775 and belinostat in patients with relapsed/refractory or poor-prognosis AML in development	[115,128]
Polo-like kinase inhibitors, e.g., BI2536, volasertib	Potentiation of DNA damage and disruption of the DNA damage response by HDACIs		[136]
Protein neddylation inhibitors (MLN4924)	Inhibition of NF-κB (activated by HDACIs) by MLN4924, ROS generation and induction of DNA damage by MLN4924 as well as by HDACIs, opposing effects on autophagy		Manuscript in preparation
BH3-mimetics, e.g., obatoclax, navitoclax, venetoclax	Up-regulation of Bim by HDACIs, which is released from Bcl-2 and Bcl-xL by ABT-737, activation of cytotoxic autophagy (obatoclax)		[189,190]
PI3K/Akt/mTOR pathway inhibitors, e.g., LY294002, buparlisib, idelalisib, duvelisib (PI3K inhibitors), perifosine (Akt inhibitor), BEZ235 (PI3K/mTOR inhibitor)	Bcl-2 and Bid cleavage, down-regulation of Mcl-1 and XIAP, MAPK/ERK inactivation, JNK activation, ROS generation, blockade of HDACI-mediated induction of p21		[160,161]

Abbreviations: HDACI, histone deacetylase inhibitor; NF-κB, nuclear factor kappa B; Hsp90, heat shock protein 90; AML, acute myeloid leukemia; CR, complete remission; SD, stable disease; DNA, deoxyribonucleic acid; ROS, reactive oxygen species; Bcl-2, B-cell lymphoma 2; XIAP, X-linked inhibitor of apoptosis; Mcl-1, myeloid cell leukemia 1; Bcl-xL, B-cell lymphoma extra long; MAPK, mitogen activated protein kinase; ERK, extracellular signal-regulated kinase; FLT3, fms-like tyrosine kinase 3; JAK2, Janus associated kinase 2; JNK, C-Jun N-terminal kinase; PI3K, phosphatidylinositol-3-kinase; mTOR, mammalian target of rapamycin; ATR, ATM (ataxia telangiectasia mutated) and Rad3-related; Chk1, checkpoint kinase 1.

**Figure 3:**
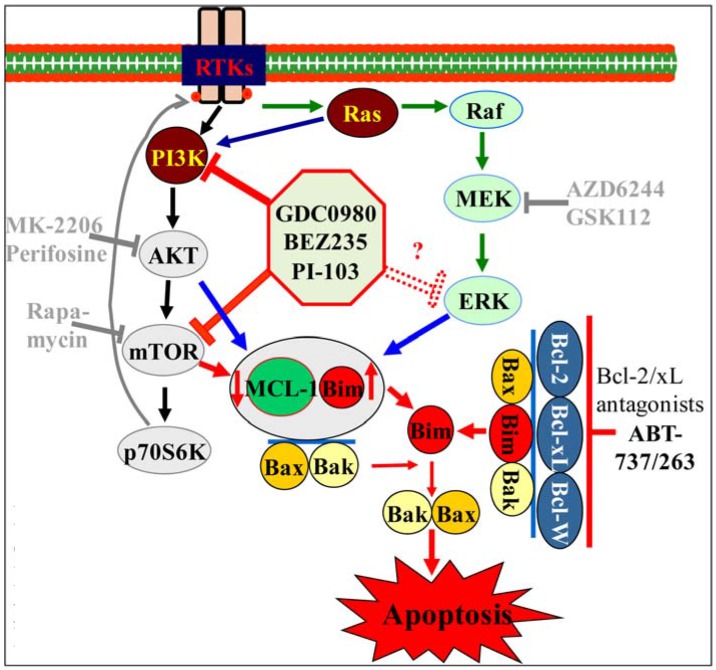
Hypothetical model of interactions between PI3K/AKT/mTOR pathway inhibitors and Bcl-2 antagonists. Reproduced, with permission, from [192].

A particularly strong rationale exists [192] for combining BH3-mimetics, e.g., ABT-737, with dual inhibitors of PI3K and mTOR, e.g., NVP-BEZ235, PI-103 or GDC-0980 in AML (Figure 3). Akt regulates a wide range of target proteins that control cellular proliferation, survival, growth and other processes, including Bim, Bad and Bax, the forkhead box O (FOXO) transcription factors (which mediate apoptosis by activating the transcription of pro-apoptotic genes such as *FasL* and *Bim*), Mdm2, glycogen synthase kinase 3 (GSK3) isoforms (which down-regulate cyclin D1 and Myc), procaspase 9, IκB kinase (the negative regulator of NF-κB), the endogenous CDKI p27^KIP1^ and Chk1 [193,194]. Importantly, mTORC1, a major downstream effector of Akt, is often not only under the control of PI3K/Akt signaling [195], and conversely, mTOR inhibition can lead to feedback activation of PI3K/Akt and MEK/ERK, arguing for simultaneous inhibition of both PI3K/Akt and mTOR [196]. Some of these agents have shown clear preclinical evidence of activity in AML [197,198]. Finally, as noted above [160,161] and in sharp contrast to other tumor types [199], in AML cells, PI3K/Akt inhibitors may disrupt, rather than activate, the complementary Ras/Raf/MEK/ERK survival signaling pathway, activated in >80% of AML samples [200], through an unknown mechanism. For these reasons, while PI3K inhibitors (e.g., GDC-0941) [201] and mTOR inhibitors plus MEK inhibitors (e.g., AZD-8055 plus selumetinib) [202] demonstrate synergistic pro-apoptotic effects with ABT-737 in AML cell lines and patient-derived blasts, accompanied by Bim up-regulation, Mcl-1 down-regulation and Bax activation, dual PI3K/mTOR inhibitors may, in fact, be the superior partner for ABT-737 [192]. Indeed, these agents (e.g., NVP-BEZ235, PI-103) synergistically increased ABT-737-mediated cell death in multiple leukemia cell lines and primary AML specimens, as well as significantly diminished tumor growth and prolonged animal survival in a subcutaneous xenograft model [203]. PI3K/mTOR inhibitors markedly down-regulated Mcl-1, apparently through a GSK3-mediated mechanism, but increased Bim binding to Bcl-2/Bcl-xL; the latter effect was abrogated by ABT-737 [203]. Responding, but not non-responding, primary samples exhibited basal AKT phosphorylation, suggesting that basal Akt activation/addiction may predict for success of this therapeutic strategy [203]. Studies are underway to see if these findings can be extended to the combination of GDC-0980 and ABT-199 (GDC-0199).

## 5. Other Rational Combinations

The PI-CDKI combination of bortezomib and alvocidib is synergistic in myeloid leukemia cells [204,205] but, to our knowledge, this combination has been tested clinically only in patients with relapsed or refractory indolent B-cell neoplasms, including MM [206,207]. In AML cells, synergistic induction of cell death was accompanied by down-regulation of Mcl-1 and XIAP, JNK activation, NF-κB inhibition, cdc2 activation and diminished expression of p21^WAF1/CIP1^ [204]. In CML cells, similar findings were noted, in addition to Bcr-Abl down-regulation, STAT3/5 inhibition and diminished phosphorylation of Lyn, Hck, CrkL, and Akt [205]. The regimen effectively induced apoptosis in imatinib-resistant cells characterized by reduced expression of Bcr-Abl but a marked increase in expression/activation of Lyn and Hck [205].

Dramatic potentiation of CDKI-induced apoptosis by inhibitors of PI3K has been demonstrated in AML cell lines and primary patient-derived blasts, accompanied by diminished Bad phosphorylation, induction of Bcl-2 cleavage, and down-regulation of XIAP and Mcl-1 [208]. In contrast, synergistic enhancement of alvocidib-induced apoptosis was not observed with inhibitors of MEK/ERK or of mTOR [208]. Much more recently, PIK-75, a compound that transiently blocks CDK7/9, leading to transcriptional suppression of Mcl-1, and also targets the p110α isoform of PI3K has been shown to rapidly induce apoptosis of AML cells, significantly reduce leukemic burden and increase the survival of mouse xenografts without overt toxicity [209].

The observation that inhibition of Chk1 triggers marked ERK1/2 activation, which can be blocked by MEK inhibitors [210] or Ras-targeting agents such as statins [211] or farnesyltransferase inhibitors [212], leading to striking increases in apoptosis and dramatically enhanced lethality, both *in vitro* and *in vivo*, along with a requirement for ERK1/2 activation in progression across the G2/M boundary and through mitosis [213], as well as functional roles for MEK/ERK signaling in the DDR [214,215] provide the rationale for combined inhibition of Chk1 and the Ras/Raf/MEK/ERK pathway in AML [90]. Furthermore, these strategies act independently of *p53* mutational status [90]. However, these early studies [210,211,212] used UCN-01 (7-hydroxystaurosporine), which functions as a CDKI and as an inhibitor of protein kinase C (PKC), in addition to inhibiting Chk1. The recent withdrawals of several investigational Chk1 inhibitors has hampered translation of this concept. However, given the modest efficacy of selumetinib monotherapy in AML [216], combined Chk1 and MEK inhibition, most recently explored in MM [217], could also warrant attention in AML.

## 6. Conclusions

The number of rational combinations of targeted agents that are possible in AML and may be effective, at least in theory, is virtually limitless. The biggest challenge, therefore, is how to most judiciously choose the most promising combinations and bring them forward into clinical trials, which are costly and time-consuming. For these reasons, consideration should also be given to identifying the biologic subtypes of AML most likely to benefit from a given combination, as illustrated by the suggestion that basal Akt activation might predict for efficacy of a strategy simultaneously targeting Bcl-2/-xL nd PI3K/mTOR. Additionally, attention needs to be paid to better trial designs, e.g., adaptive designs, to get us answers to the biggest challenges confronting our patients in the most expeditious manner possible. Finally, the current paradigm for regulatory approval of new drugs in the United States discourages manufacturers from venturing into combinations of unapproved agents, and slows the pace of therapeutic progress. As a result, very few of the combinations discussed in this article have been tested in patients. While a complex problem, this is one that will require a concerted effort by lawmakers, researchers, industry and the concerned public to effect real change in the fight against cancer in general, and AML in particular.
